# Macromolecular Crowding Regulates the Gene Expression Profile by Limiting Diffusion

**DOI:** 10.1371/journal.pcbi.1005122

**Published:** 2016-11-28

**Authors:** Mahdi Golkaram, Stefan Hellander, Brian Drawert, Linda R. Petzold

**Affiliations:** 1 Department of Mechanical Engineering, University of California, Santa Barbara, California, United States of America; 2 Department of Computer Science, University of California, Santa Barbara, California, United States of America; Purdue University, UNITED STATES

## Abstract

We seek to elucidate the role of macromolecular crowding in transcription and translation. It is well known that stochasticity in gene expression can lead to differential gene expression and heterogeneity in a cell population. Recent experimental observations by Tan *et al*. have improved our understanding of the functional role of macromolecular crowding. It can be inferred from their observations that macromolecular crowding can lead to robustness in gene expression, resulting in a more homogeneous cell population. We introduce a spatial stochastic model to provide insight into this process. Our results show that macromolecular crowding reduces noise (as measured by the kurtosis of the mRNA distribution) in a cell population by limiting the diffusion of transcription factors (i.e. removing the unstable intermediate states), and that crowding by large molecules reduces noise more efficiently than crowding by small molecules. Finally, our simulation results provide evidence that the local variation in chromatin density as well as the total volume exclusion of the chromatin in the nucleus can induce a homogenous cell population.

## Author Summary

The cellular nucleus is packed with macromolecules such as DNAs and proteins, which leaves limited space for other molecules to move around. Recent experimental results by C. Tan *et al*. have shown that macromolecular crowding can regulate gene expression, resulting in a more homogenous cell population. We introduce a computational model to uncover the mechanism by which macromolecular crowding functions. Our results suggest that macromolecular crowding limits the diffusion of the transcription factors and attenuates the transcriptional bursting, which leads to a more homogenous cell population. Regulation of gene expression noise by macromolecules depends on the size of the crowders, i.e. larger macromolecules can reduce the noise more effectively than smaller macromolecules. We also demonstrate that local variation of chromatin density can affect the noise of gene expression. This shows the importance of the chromatin structure in gene expression regulation.

## Introduction

Even in an isogenic cell population under constant environmental conditions, significant variability in molecular content can be observed. This variability plays an important role in stem cell differentiation [1], cellular adaptation to a fluctuating environment [2], variations in cellular response to sudden stress [3], and evolutionary adaptations [4]. However, it can also be detrimental to cellular function and has been implicated as a factor leading to dangerous diseases such as haploinsufficiency [5], cancer [6], age-related cellular degeneration, and death in tissues of multicellular organisms [7]. The variability stems both from stochasticity inherent in the biochemical process of gene expression (intrinsic noise) and fluctuations in other cellular components (extrinsic noise), namely, stochastic promoter activation, promoter deactivation, mRNA, and protein production and decay, as well as cell-to-cell differences in, for example, number of ribosomes [8–13]. One consequence of biological noise in gene expression is transcriptional bursting, which is observed in both prokaryotes [14] and eukaryotes [12, 15]. Transcriptional bursting can bring about a bimodal distribution of mRNA abundance in an isogenic cell population [16–18]. Therefore, understanding critical factors that influence noise in gene expression can provide us with a new tool to tune cellular variability [19–24].

The cellular environment is packed with proteins, RNA, DNA, and other macromolecules. It is estimated that 30–40% of the cell volume is occupied by proteins and RNA [25]. Macromolecular crowding has been studied extensively in the last few decades [26, 27] and has been ingeniously utilized for numerous medical purposes [28–30]. It is well established that macromolecular crowding can reduce diffusion rates and enhance the binding rates of macromolecules [31], which can change the optimal number of transcription factors [32], the nuclear architecture [33], and the dynamical order of metabolic pathways [34].

It is known that manipulating the binding and unbinding rates (*k*_on_ and *k*_off_) can affect the likelihood of observing transcriptional bursting [42, 43]. Higher values of *k*_on_ and *k*_off_ lead to a bimodal distribution and transcriptional bursting, while keeping the basal (i.e. in the absence of the bursts) protein abundance constant. It is also known that macromolecular crowding can alter diffusion and reaction rates [44, 45]. Together, it is implied that macromolecular crowding can have an impact on protein production in a cellular environment.

In a previous study [35], crowding has been modeled by the direct manipulations of reaction rates using experimentally fitted relations. In contrast, we model macromolecular crowding explicitly by altering the effective diffusion rate of transcription factors. This approach is similar to recent studies performed by Isaacson *et al*. [46] and Cianci *et al*. [51]; however, we also consider the effects of the artificial crowding agents, in order to capture analogous experimental conditions performed by Tan *et al*. [35].

It has been observed experimentally [35] that macromolecular crowding can influence cell population homogeneity and gene expression robustness. In this experiment [35], the influence of the diffusion of macromolecules on transcriptional activity is studied by synthesizing artificial cells in which inert dextran polymers (Dex) assume the role of the artificial crowding agent in the system. To capture the impact of the size of the crowding agent, the experiments are performed on two different sizes of Dex molecules, here referred to as Dex-Big and Dex-Small. It can be inferred from this experiment that a highly crowded environment results in a narrow distribution of fold gene-expression perturbation, suggesting that molecular crowding decreases the fluctuation of gene expression rates due to the perturbation of gene environmental factors.

However, the mechanism by which cellular crowding can control gene expression has not been elucidated. We demonstrate through modeling that macromolecular crowding reduces the noise (kurtosis of the mRNA distribution) in gene expression by limiting the diffusion of the transcription factors. This increases the residence time of the transcription factor on its promoter, thereby reducing the transcriptional noise. As a consequence, unstable intermediate states of gene expression pattern will diminish. Furthermore, our model reveals that small crowding agents reduce noise less than large crowding agents do, which is in agreement with the experimental observations [35]. Finally, our simulation results provide evidence that local variation in the chromatin density, in addition to the total volume exclusion of the chromatin in the nucleus, can alter gene expression patterns.

## Results

A simple and well-studied model was employed to simulate transcription and translation. The model includes: a) one transcription factor (TF) placed randomly in the simulation domain, b) TF diffusion in order to find the gene locus, c) binding and unbinding of TF to its promoter, d) mRNA production, and destruction and e) protein production and destruction. This model and its corresponding parameters were adopted from Kaeren *et al*. [8] for the sake of comparison. (The details of the model are available in Materials and Methods).

We assume that the initial concentrations of mRNA and the target protein are zero, and use spatial stochastic simulation to investigate the gene expression pattern, a model that has been widely used and verified by both theoretical [36–39] and experimental [39, 40] observations. To account for crowding, we developed a modified next subvolume method (NSM) to approximately solve the reaction-diffusion master equation (RDME) [41] capable of explicitly treating the crowding agent amount, distribution, and interactions (Materials and Methods).

The NSM method was modified so that the mesoscopic diffusion coefficient is linearly dependent on the crowding density in the destination voxel. In our model, the macromolecular crowding stems from two primary sources: chromatin structure and artificial crowding agents, akin to the Tan *et al*. experiment [35]. We utilized the 3-dimensional structured illumination microscopy data from [50] to model the chromatin structure. To account for chromatin structure, the crowding density in each voxel was assumed to be proportional to DAPI (4',6-diamidino-2-phenylindole) intensities in that voxel, similar to the method introduced by Isaacson *et al*. [46]. To account for different levels of crowding, we added artificial crowding agents distributed randomly in our simulation domain. We define the crowdedness parameter θ as the probability for each voxel to be occupied by an artificial crowding agent. Thus, we are able to explicitly account for different amounts of crowding in our simulation domain by changing θ. To interpret θ correctly, let`s consider the extreme case where θ = 1. In this case all voxels would be occupied by one and only one crowding agent. Then, crowding reduces the diffusion coefficient depending on the size of the crowding agent (90% reduction for a large crowder vs. 40% reduction for a small crowder.). Note that under no condition would any voxel be completely blocked (i.e. 100% crowded). For any other θ, approximately θ×N crowding molecules are randomly distributed in θ×N voxels, where N is the total number of the voxels. A convergence study demonstrates that our conclusions are independent of voxel size for a sufficiently small mesh, see (S4 Fig).

To validate the model, the simulation was run for 1000 minutes with the same parameters as in [8] while θ = 0, i.e. with no artificial crowding agent or chromatin present. As in [8], this resulted in transcriptional bursts. A direct quantitative comparison is not trivial due to the fact that our model is spatially inhomogeneous (S2 Fig).

**Fig 1:**
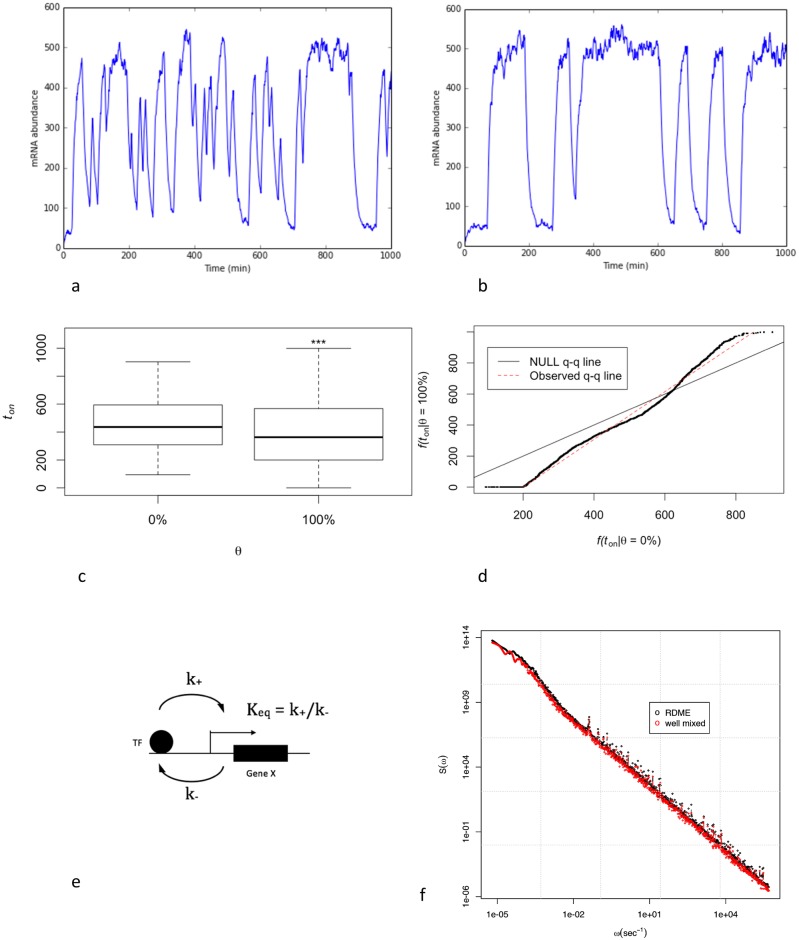
Macromolecular crowding can increase the transcriptional bursting by limiting the diffusion and increasing the residence time of TF on the promoter. a) gene expression dynamics in the presence of chromatin as the only volume exclusion factor. b) gene expression dynamics when a large crowding agent (e.g. Dex) is added. c) comparison between the distributions of active state duration (*t*_*on*_) for θ = 0% vs. θ = 100% (*** p-value < 0.001). d) qq-plot of distributions of *t*_*on*_ for θ = 0% vs. θ = 100%. Significant deviation between quantiles of *t*_*on*_ distributions (fitted red line) and the Null distribution implies a reduction in average *t*_*on*_. e) effective two states well-mixed gene model. c and d are obtained by 3200 trajectories of 1000 min simulations. f) the power spectrum of mRNA obtained by the spatial model is in agreement with a well-mixed model using the effective rate constants.

Next, we included the artificial crowding agent and the chromatin in our model and investigated mRNA abundance in our simulation domain for low and high θ values (θ = 0% vs. θ = 100%). It can be seen that the system switches more frequently between active and inactive states for low θ values than it does for high θ values (Fig 1a and 1b). We hypothesized that adding the artificial crowding agent limited the diffusion of the TF. Thus, the TF tends to stay in either of the two stable states (active or inactive states) for a longer period of time. This increase in the residence time of the TF on the promoter results in reduced transcriptional bursting.

Next we studied the effect of the artificial crowding agent on biochemical rates, by comparing the distributions of active state duration (*t*_*on*_) for 3200 trajectories of 1000 min simulations (Fig 1c and 1d). Fig 1c and 1d show a significant (p-value < 0.001) decrease in *t*_*on*_ for θ = 100%. Likewise, given *t*_*on*_ + *t*_*off*_ = 1000 min, we observe a significant increase in *t*_*off*_ for θ = 100%. Therefore, using gene activation rate constant k_+_ ~ < *t*_*off*_>^-1^ (<.> denotes the mean), our simulation results suggest a 12% decrease in k_+_ (in agreement with [65]) and a 23% increase in the deactivating rate constant (k_-_). Our model predicts a smaller reduction in k_-_ compared to [65], and thus, predicts a 29% decrease in equilibrium constant (K_eq_ = k_+_/k_-_) whereas [65] predicts an increase in K_eq_. This discrepancy might be due to the assumption in [65] that the association rates are always diffusion limited. It would be interesting to repeat similar simulations using particle level methods such as molecular dynamics to obtain a more precise estimate of the change in the equilibrium constant. Our finding is in qualitative agreement with the experimental observation that a crowded condition of heterochromatin can repress gene expression [49] (Fig 1e).

Van Paijmans and Ten Wolde [60] showed that in general the abovementioned biochemical system can be reduced to a well-mixed model if there is a clear separation of time scales between rebinding and binding of molecules from the bulk, which can be deduced from the power spectrum of the mRNA expression. Briefly, a characteristic knee in the low-frequency regime (corresponding to Markovian switching at long times), which is well separated from the regime corresponding to the rebindings at higher frequencies renders it possible for a spatially resolved biochemical system to be reduced to a well-mixed system. To explore whether our system can be reduced into a well-mixed system, we used the effective biochemical rate constants obtained by measuring the transcriptional activity (Fig 1e). By comparing the power spectrum of the spatial model for the special case when θ = 100% with the corresponding well-mixed model, we conclude that once the effective biochemical rate constants are measured using our spatial model for a given configuration (i.e. distinct crowding size and distribution), spatial model can be reduced into a well-mixed model (Fig 1f). Note, however, as shown later in this study, these biochemical rate constants depend strongly on the size and the distribution of the crowding agent molecules, and the local chromatin density. Hence, a spatial model is required to measure these constants.

**Fig 2:**
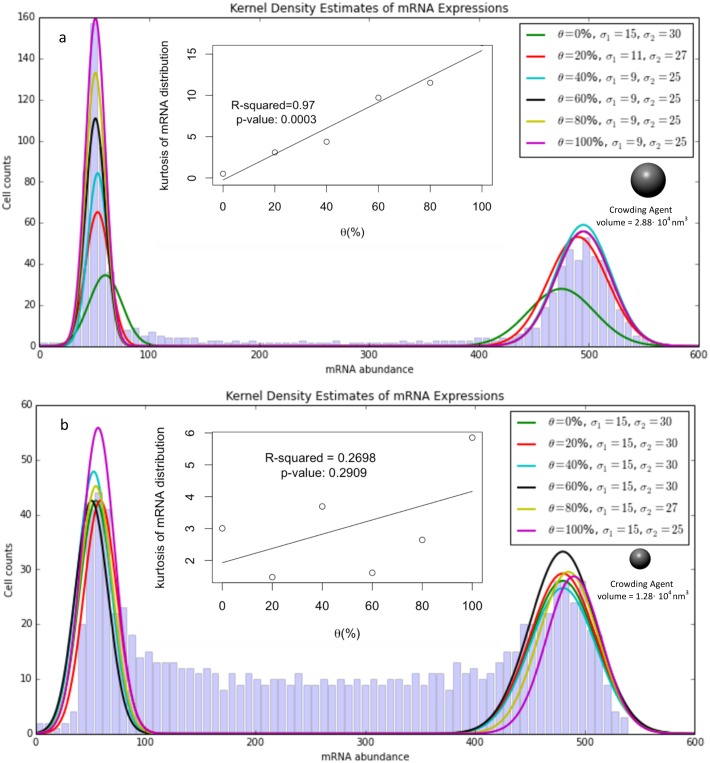
a) A large crowding agent can homogenize a cell population effectively. Adding a crowding agent diminishes the probability of the intermediate states (i.e. 100<mRNA<400) and results in a more uniform cell population that lies in either of the two stable states (p-value < 0.01). The results illustrate a strong correlation between the concentration of the crowding agent and the kurtosis of the distribution of mRNA abundance. Each distribution represents 16000 data points and was obtained using kernel-density estimate (KDE) [52]. b) A small crowding agent is incapable of producing a uniform cell population (p-value > 0.01). Intermediate states remain intact after crowding the cells by a small crowding agent. Each distribution represents 16000 data points and was obtained using kernel-density estimate (KDE). The histograms show the total number of the cells with certain expression levels. Here only histograms for θ = 100%, large crowding agent (a) and θ = 0% (b) which provide the least and the most intermediate states are shown. σ_1_ and σ_2_ are the approximate standard deviation corresponding to the first and the second mode, respectively.

To analyze the consequences of macromolecular crowding on a cell population, we simulated 16000 isogenic cells in an analogous situation for different values of θ. We observed (Fig 2a) that while low θ values can diversify the cell population and result in intermediate states (two peaks correspond to two stable states, i.e. active and inactive states), with higher values of θ we observed a more homogeneous population (no intermediate states). This observation is in agreement with recent experimental results [35]. In this situation, the average number of mRNA is close to the number of mRNA obtained when noise is removed from gene expression (deterministic models). Our simulation results show that adding the crowding agent to the simulation domain replaces the intermediate states by two more stable states. The two stable modes (mRNA abundance = 50 and 500) are intact after crowding the simulation domain (Fig 2a). It can be inferred from our linear model that there is a statistically significant correlation between kurtosis of the mRNA distribution and the amount of the crowding agent (p-value < 0.01).

We should stipulate that the kurtosis values define the noise in our system. Low kurtosis values correspond to a cell population in which mRNA expression in each cell is near either the first or the second peak (i.e. ~50 and 500). Conversely, high kurtosis corresponds to a cell population in which certain cells have mRNA expression levels that lay between the peaks (i.e. intermediate states). Likewise, a more homogenous cell population can be obtained by removing the intermediate states (i.e. higher kurtosis value and narrower distributions or lower noise).

It has been observed experimentally [35] that the larger crowding agents (Dex-Big) can contribute robustness to the gene expression pattern more effectively than the smaller crowding agents (Dex-Small). To examine whether our model would reproduce this observation, we repeated the previous simulations using smaller crowding agents (~2 times smaller by volume fraction). Larger crowding agents occupy more volume in a voxel and reduce the diffusion coefficient more effectively than smaller crowding agents (90% reduction in the diffusion coefficient for larger crowding agents compared to 40% reduction for smaller crowding agents). However, by occupying more voxels (~2 times as many voxels as in the larger crowding agent case), a similar level of volume exclusion can be achieved by smaller crowding agents. Note that in order to assess the effect of the artificial crowding agent size, one should compare the kurtosis of mRNA distributions for θ values that correspond to similar total volume exclusion for Dex-Big vs. Dex-Small (e.g. Dex-Big and θ = 60% vs. Dex-Small and θ = 100%).

Our diffusion-limited gene expression model is capable of reproducing the same experimental observations (Fig 2b). Our simulation results suggest that the intermediate states do not vanish, despite adding a substantial amount of small crowding agents. Our linear regression model illustrates a small correlation between the kurtosis of the mRNA distribution and the amount of the crowding agent (p-value > 0.01). Therefore we can conclude that, in agreement with experimental observations, our model shows that the smaller crowding agents cannot homogenize the cell population effectively. This is not surprising since small molecules exist in the cellular environment in high concentrations but their impact on gene expression is negligible compared to histones, mRNAs and regulatory proteins.

**Fig 3:**
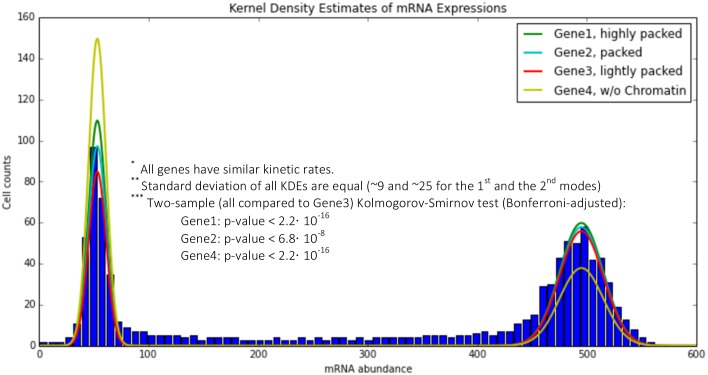
Effect of the chromatin structure on the gene expression pattern. Not only does the chromatin structure reduce the diffusion rate due to the macromolecular crowding effect, it also can determine the transcription pattern of different genes due to their location. Each distribution is compared to gene 3 (red curve) using Kolmogorov-Smirnov test (Bonferroni-adjusted for multiple comparisons). p-values show significant difference between distributions (each distribution is obtained by 16000 data points). Histograms show the total number of the cells with certain Gene2 expression levels, indicating the intermediate states for this gene.

Next, we analyzed the impact of chromatin reorganization, to understand how the local volume exclusion of chromatin can influence the gene expression patterns of specific genes. Three different genes were selected (Genes 1–3) to account for super dense (Gene 1), dense (Gene 2) and sparse chromatin area (Gene 3). Identical model and simulation parameters were used for all three genes to control for other effects except the volume exclusion of chromatin. By comparing the mRNA distributions of cell populations consisting of 16000 cells, our simulation results suggest that diffusion-limited gene expression can alter mRNA production in a cell population (Fig 3). Here, the two-sample (all compared to Gene3) Kolmogorov-Smirnov (KS) test (Bonferroni-adjusted) was used to compare different mRNA distributions and a statistically significant difference was obtained (p-value < 0.01).

**Fig 4:**
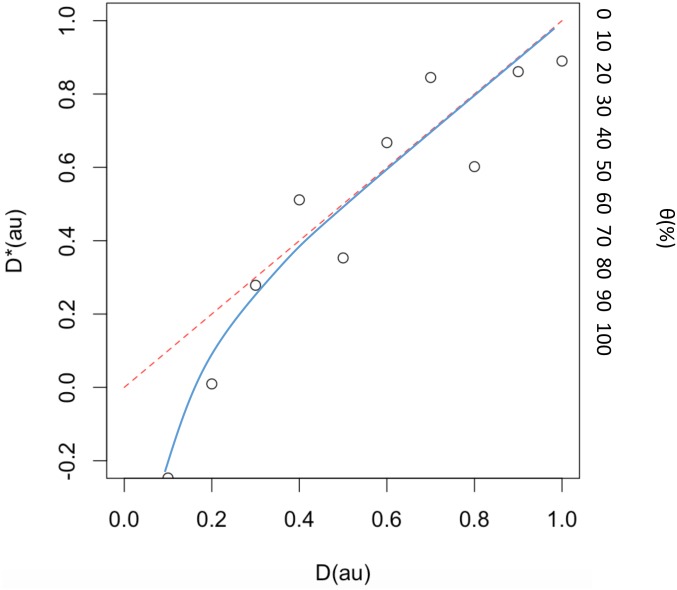
Impact of the diffusion-limited gene expression on the cell population diversity (D* is the effective diffusion coefficient). The red dotted line shows the hypothetical line that explains the variability in a cell population completely as a result of diffusion-limited gene expression. The blue line is a logarithmic fit to our simulation results and supports the hypothesis of robust gene expression as a result of diffusion-limited gene expression (a large crowding agent was used).

To demonstrate that macromolecular crowding reduces the gene expression noise primarily by volume exclusion, thus limiting the diffusion, we repeated the simulations in the absence of the crowding agents but using different diffusion coefficients. This was implemented by replacing the diffusion coefficients (D) with the effective diffusion coefficient (D*) (Materials and Methods). Each data point (X, Y) in Fig 4 was found by running the simulation for different D values (X) and evaluating the kurtosis of mRNA distributions. Then the corresponding D* values (Y) were obtained by Eq 3. We hypothesized that if macromolecular crowding is capable of reducing the noise of gene expression primarily by slowing down the diffusion of TF, we should expect to see a linear fit in our data points with the hypothetical line (Fig 4, red dotted line). As shown in Fig 4 our simulation results support this hypothesis for a physical range of θ values (0–100%), for a large crowding agent.

As previously discussed, the size of the crowding agent plays a vital role in obtaining a homogeneous cell population. By comparing the kurtosis values of the mRNA distributions obtained using a large crowding agent (θ = 60%) vs. a small crowding agent from Fig 2 (θ = 100%), where the total volume exclusion is similar, different phenotypes can be observed (kurtosis value of ~10 vs. ~4). Furthermore, the position of the gene within the chromatin matters. It can be inferred that although the overall volume exclusion effect is similar for all three genes, the local chromatin density can alter the time a TF requires to reach its target. In sum, our study suggests that macromolecular crowding can influence the gene expression noise significantly, both locally and globally (Fig 3, yellow curve, p-values < 0.01).

## Discussion

A significant portion of cell volume is occupied by proteins, RNAs and other macromolecules. To obtain a complete understanding of the pattern of gene expression, a comprehensive understanding of the impacts of macromolecular crowding is essential. In this study, we have proposed a simple model similar to that of [46] to account for macromolecular crowding in the cellular environment. We utilized the NSM method for simulation of the reaction-diffusion master equation, to include macromolecular crowding. We have avoided any direct manipulation of reaction rates to account for macromolecular crowding [35]. In addition, our method facilitates an explicit treatment of macromolecular crowding, in that geometric dependency of chromatin structure on gene expression is addressed, and interactions between the crowding agent and different molecules can be considered. This provides a platform to assess how the chromatin structure impacts gene expression. Our model accounts for the addition of the artificial crowding agent and its size, and demonstrates that macromolecular crowding can homogenize a cell population by limiting the diffusion of TFs. Therefore, it improves our understanding of the underlying sources of gene expression noise from that of the earlier models [35, 46].

Our model predicts that a large crowding agent (Dex-big), reduces the diffusion coefficient of TF more effectively than a small crowding agent (Dex-small), in agreement with the experimental observations by Tan *et al*. Likewise, it can be inferred from other experimental observations by Phillies *et al*. [69] that the molecular weight and concentration of crowding molecules can change the diffusion coefficient considerably, whereas the size of a TF has insignificant impact. Finally, although Muramatsu and Minton [68] observed an inverse correlation between the size of the crowder and that of the diffusion coefficient, Phillies *et al*. [69] has shown the opposite (this controversy is discussed in [68] as well).

It is worth noting that Isaacson *et al*. [46] used spatial stochastic simulation to show that the first passage time (the time required for TF to find the gene locus) decreases to a minimum at first, and then increases again as the volume exclusion due to chromatin increases further. That study suggests that crowding can accelerate or decelerate the diffusion depending on the density of the crowding agent, leading to faster or slower chemical kinetics, respectively. Our study, on the other hand, demonstrates the mechanism by which crowding can reduce the transcriptional noise of gene expression. For an intuitive understanding of the gene expression noise reduction mechanism, first note that as shown by van Zon *et al*. [47], TF diffusion is the dominant source of gene expression noise. Also, macromolecular crowding can effectively partition the available space into smaller compartments, which not only linearizes the input–output relation, but also reduces the noise in the total concentration of the output. In fact, by partitioning the space, macromolecular crowding isolates molecules, as a result of which the molecules in the different compartments are activated independently, thereby reducing the correlations in the gene expression switch. Consequently, this removal of correlations can lower the output noise [48]. We suggest the following function for the macromolecular crowding, by which a uniform cell population can be obtained. By comparing Fig 1a and 1b, it can be inferred that macromolecular crowding can increase the average residence time of TF on its promoter. As a consequence, transcriptional bursts are attenuated which leads to elimination of the intermediate states in the mRNA distributions.

Our findings demonstrate the importance of spatial simulations to fully capture several experimental observations. Morelli *et al*. [65] studied the effect of macromolecular crowding on a gene network by rescaling the association and dissociation constants into a well-mixed model. Here, on the other hand, we provide strong evidence (Figs 2 and 3 and S4 Fig) that the impact of crowding structure and distribution cannot be fully understood using well-mixed models.

Furthermore, our model sheds light on how to develop engineered cells to achieve advantages in gene expression, cellular computing and metabolic pathways [35]. Investigations of other epigenetic factors show that DNA methylation and chromatin structure may be linked to transcriptional activity, both in single cells and across populations. Gene silencing by histone modification or formation of repressed chromatin states (heterochromatin) are good examples of how nature has exploited macromolecular crowding and inherent stochasticity in gene expression to display new traits [49]. Our methodology can be utilized to further assess heterochromatin and euchromatin functional differences at a reasonable resolution.

## Materials and Methods

We used a well-known, simple model to describe transcription and translation [8]. Transcription factor (TF) was added to that model to account for spatial effects of TF diffusion in a crowded environment. Given a cubic domain in which protein production takes place, gene expression begins by TF diffusion and finding the locus of the gene of interest. Upon binding/unbinding of TF to/from its promoter, the gene switches between active and inactive states. Without loss of generality, the gene of interest is placed in the center of a box with a characteristic length L. One and only one TF can activate the promoter. Thus, the chemical system of protein production can be written as:

$$TF+Promoter_{Repressed}\begin{array}{c} k_{on},\;k_{off}\\ ⇌ \end{array}Promoter_{Active}\overset{s_{A}}{\to }M+Promoter_{Active}\;(\text{R}1)$$

$$Promoter_{Repressed}\overset{s_{R}}{\to }M+Promoter_{Repressed}\;(\text{R}2)$$

$$M\overset{s_{P}}{\to }P+M\;(\text{R}3)$$

$$M\overset{\delta_{M}}{\to }\emptyset \;(\text{R}4)$$

$$P\overset{\delta_{P}}{\to }\emptyset \;(\text{R}5)$$

The simulation parameters were adopted from [8] for the sake of comparison with non-spatial methods (Table 1).

**Table 1:** Modell Parameters.

Parameters	Values	Reference	Species	Initial Conditions
*D*_*TF*_	10 (μm^2^.min^-1^)	[53]	Transcription Factor (TF)	1
*D*_*Chromatin*_	~ 0	[54]	mRNA (M)	0
k_on_	0.1 (min^-1^)	[55]	Protein (P)	0
k_off_	0.1 (min^-1^)	[56]	Simulation Parameters	Values
s_A_	50 (min^-1^)	[8]	Box Dimensions [35]	1μm by 1μm by 1μm
s_R_	5 (min^-1^)	[8]	Number of voxels	50 by 50 by 11
s_P_	0.2 (min^-1^)	[8]	Simulation Time	1000 min
_P_	0.05 (min^-1^)	[8]		
δ_M_	0.1 (min^-1^)	[8]		

### Simulation algorithm

The inherent stochastic characteristics of gene expression, along with the failure of deterministic models to produce transcriptional bursting, lead us to consider a spatial stochastic model. A modified next subvolume method (NSM) [41] was used to simulate the stochastic reaction-diffusion system, using the implementation in PyURDME on the MOLNS software platform [57]. We developed the following modifications to account for crowding (for access to the software implementation, see URL in [58]).

Inside the cell, the chromatin, histones, etc., are crowding the nucleus. Note that we are ignoring dynamic addition and reduction of newly synthetized proteins (*P*) and mRNAs (*M*) since they are negligible when compared to the chromatin. Given a domain which is discretized into N uniform voxels, each voxel is occupied with the artificial crowding agent with a probability θ. The diffusion between two adjacent voxels is linearly dependent on the crowding density of the destination voxel, consisting of the chromatin and the artificial crowding agent. This model assumption is analyzed in detail and compared with the available experimental data in Supporting Information (S1 Fig). This model does not explicitly take into account lock-in effects, that crowding in the origin voxel may affect the diffusion rate to adjacent voxels, or that the effective reaction rate in a voxel may depend on the local crowding and configuration of the chromatin and crowders. For instance, Friedman [66] showed that hydrodynamic effects cause a 15% reduction in the computed rate constant for neutral species or ions in water. The impacts of the electrostatic forces have been widely studied and considered primarily in molecular level simulations [67]. Specific chromatin configurations can affect the hopping rate of particles differently. Namely, even in low chromatin concentrations, distinct configurations might be able to fully trap the particle and reduce the hopping rates significantly. However, we believe that our model is sufficiently accurate to study the qualitative effects of crowding.

It is worth mentioning that our model ignores any non-specific interaction between DNA and TF. Paijmans and ten Wolde [60] showed quantitatively that even in the presence of 1D sliding along the DNA, which makes rebinding events not only more frequent but also longer, the effect of diffusion can still be captured in a well-stirred model by renormalizing the rate constants. However, renormalization does not account for the architecture of chromatin and how it can influence the rate constants. Although several studies suggest that such non-specific interactions can help TF to slide on the DNA strand (facilitated diffusion) to find the target faster [61, 62], recent work by Wang F *et al*. [63] provides evidence that the promoter-search mechanism of E. coli RNAP is dominated by 3D diffusion. Moreover, in another work [64] the sliding length of TF on DNA is measured to be ~30–900 bps. In our simulation, on the other hand, each voxel contains ~Mbps and therefore, on the length scale of our model, facilitated diffusion is insignificant.

The size of the crowding agent is modeled by the parameter δ_*i*_. We assume that smaller crowders reduce the diffusion less than large crowders. In our simulations we let *δ*_*i*_ = 0.6 for smaller crowding agents, *δ*_*i*_ = 0.1 for larger crowding agents, and *δ*_*i*_ *=* 1 when no crowding agent is present. Thus, the diffusion rate into voxel *i* is computed as

$$D=D_{0}\times (1-c_{i})\times \delta_{i}$$ (1)

where *c*_*i*_ models the crowding due to the chromatin in voxel *i*. It is unknown exactly how the concentration of chromatin affects the effective diffusion, but as a simple model we assume that

$$c_{i}=\frac{DAPI\;intensity\;in\;cell\;i}{max_{j}\;DAPI\;intensity\;in\;voxel\;j}$$ (2)

The diffusion rate thus depends linearly on the DAPI intensity, and we assume that the voxel with the highest intensity of DAPI is fully blocked. For simplicity we assume that neither the chromatin nor the crowding agent diffuses between voxels.

The TF molecule is initially placed randomly in the domain. During the simulation it will diffuse to the gene locus and activate transcription. Recent studies [46, 51] have proposed more complicated relations to obtain the effective diffusion coefficient in the presence of macromolecular crowding. Here, we use a linear relation to calculate the TF diffusion coefficient as a function of the total crowdedness (i.e. the effects of both chromatin structure and artificial crowding agents included). This simple relation can capture physiologically relevant trends and suffices for the purpose of our simulations.

### Effective diffusion rate calculation

Considering the total effect of the artificial crowding agent as

$$D^{*}=\frac{\sum_{i}\delta_{i}}{N}D,$$ (3)

where *i* is the voxel index and *N* is the total number of voxels in the domain. For a large crowding agent, Eq 3 leads to D* = [θ×0.1 + (1- θ) ×1]D = (1–0.9 θ)D. Using the linear model presented in Fig 2a (Kurtosis(θ) = 15 θ), we obtain (for D = 1)

D* =1− 0.06 × Kurtosis (4)

to calculate the effective diffusion rate. Each data point (X, Y) in Fig 4 is found by running the simulation for different D values (X) and evaluating the kurtosis of the mRNA distributions. Then the corresponding D* values (Y) are obtained by Eq 4.

In summary, in order to obtain the effective diffusion as illustrated in Fig 4, the following procedure has been followed:

1. We performed different simulations by varying the diffusion coefficient *D* in the absence of any crowding, and the kurtosis values of mRNA distributions were calculated.
2. Next, in order to pinpoint the corresponding effective diffusion D* that leads to the same kurtosis value as *D* in the presence of a crowder, we need to determine the crowding parameter (θ). θ can be estimated using a linear regression model as shown in Fig 2a, Kurtosis(θ) = 15 θ.
3. Finally, by substituting θ into D* = [θ×0.1 + (1- θ) ×1]D = (1–0.9 θ)D from Eq 3, D* can be obtained using Eq 4.
4. 1–3 should be applied to all D values to obtain a set of (D, D*) in order to produce Fig 4.

### Statistics

All statistical tests were performed using the ‘R’ statistics package, an open-source software package based on the ‘S’ programming language (http://www.R-project.org). All correlations were calculated using the Pearson’s product-moment correlation coefficient. Comparisons between multiple distributions were undertaken using the two-sample Kolmogorov-Smirnov test corrected for multiple testing with Bonferroni Method.

### Image analysis

All image analysis tasks were performed using ImageJ. Each of the nine stacks was discretized using a 50 by 50 Cartesian mesh, and the DAPI intensity of each voxel was measured using ImageJ [59].

### PyURDME software source code

https://github.com/mgolkaram/pyurdme/tree/crowding

## Supporting Information

S1 Text Supplementary text includes analysis of transcriptional bursting in the absence of macromolecular crowding, translational bursting and study of voxel size effect. (PDF)
