# Supplementary material for: Macromolecular Crowding Regulates the Gene Expression Profile by Limiting Diffusion
Source: PLoS Comput Biol. 2016 Nov 28;12(11):e1005122. doi: 10.1371/journal.pcbi.1005122 (PMC5125560; doi:10.1371/journal.pcbi.1005122)
Supplement: S1 Text — (PDF) [file pcbi.1005122.s001.pdf]

Supporting Information

Impact of macromolecular crowding on the diffusion coefficient

To model the impact of crowding on the diffusion coefficient, we assume a linear relation between the diffusion coefficient and the concentration of the crowding. Given Eq 5, we have

$$\frac{D}{D_0} = (1 - c_i) = 1 - \frac{I_i}{I_{max}} = 1 + (-\frac{1}{I_{max}})I_i \qquad (S1)$$

where I_i is the DAPI concentration in voxel i. Comparison between Eq S1 for the right choice of the normalization factor I_{max} and the available experimental data for Hemoglobin [1] over physiological crowding ranges shows a good agreement between the model assumption and the experimental observations (S1 Fig).

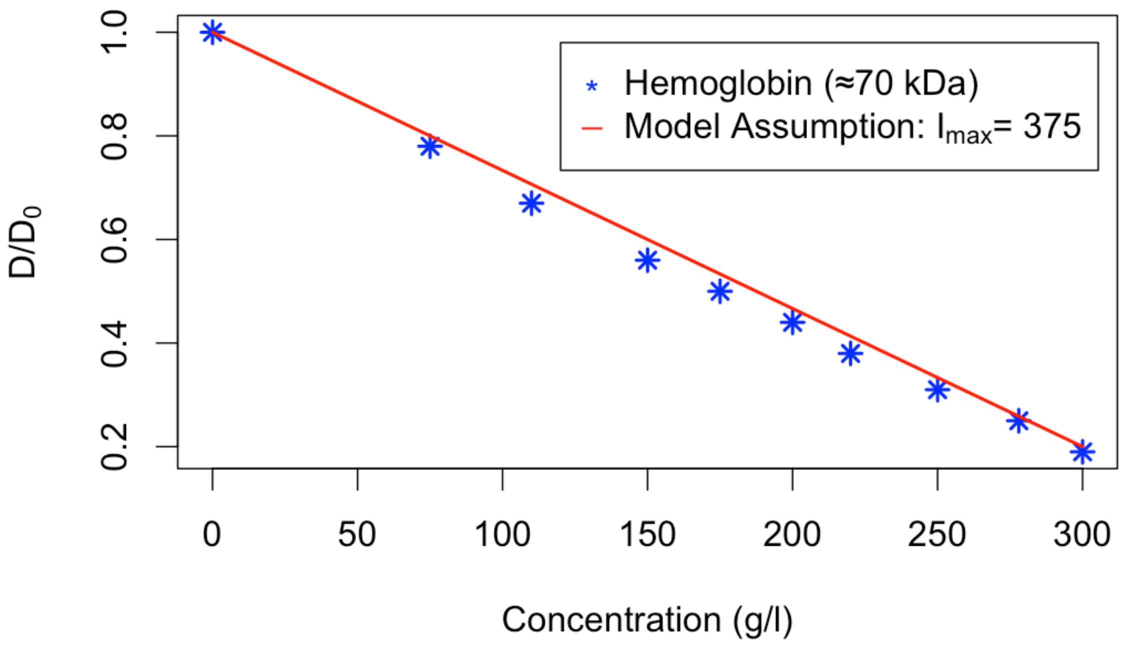

S1 Fig. The validity of a linear relation between the diffusion coefficient and the crowder concentration over physiological crowding ranges.

Transcriptional bursting in the absence of macromolecular crowding

To verify our model with the previous non-spatial stochastic simulations [2], gene expression in the absence of the crowding agent and the chromatin structure (i.e. $D_i = D_0$) was studied. It can be seen (S2 Fig) that our model is capable of producing transcriptional bursting similar to that reported for well-mixed stochastic simulation results [2].

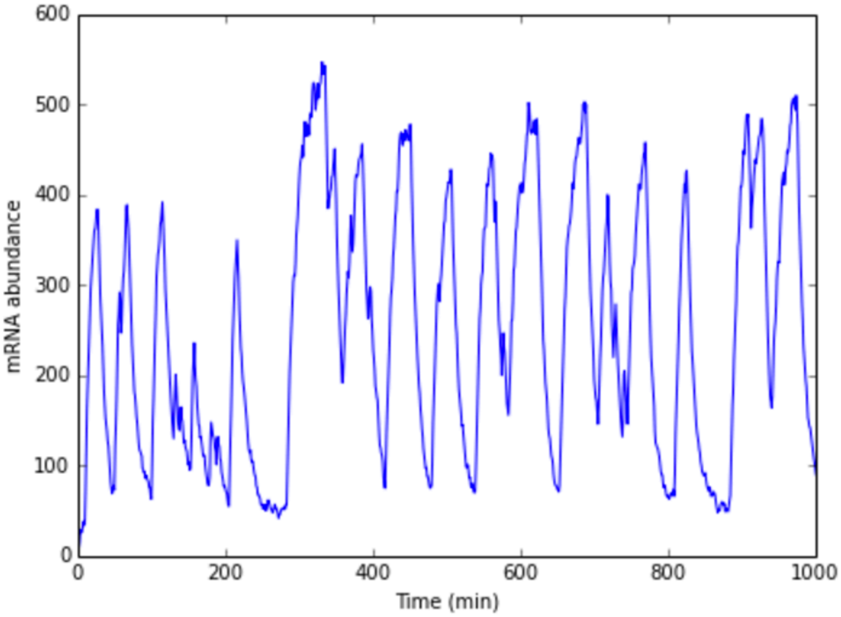

S2 Fig. Transcriptional bursting in the absence of macromolecular crowding.

Impact of Macromolecular Crowding on Translational Bursting

To assess how translational bursting can be affected by macromolecular crowding, we present a quantitative description of noise for different values of crowdedness (S3 Fig). Our analysis suggests that macromolecular crowding has an insignificant impact on translational bursting (standard deviation (SD) is used to compare the gene expression noises).

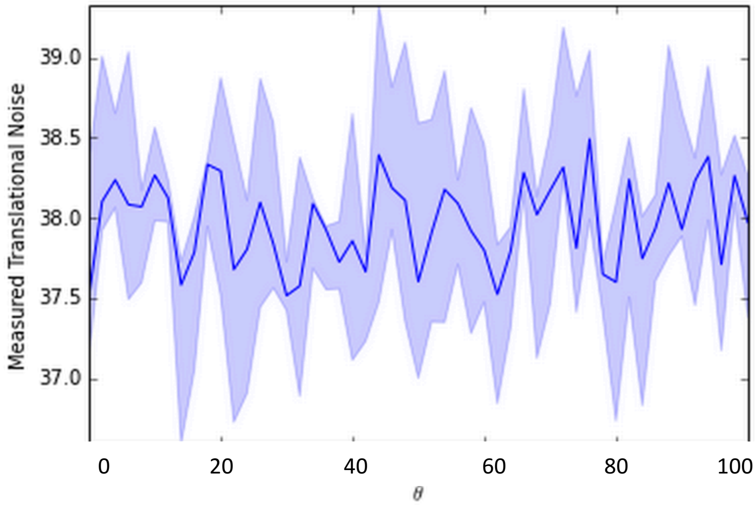

S3 Fig. Comparison between translational bursting and gene expression noise for various amounts of crowding agents. Macromolecular crowding has an insignificant impact on translational bursting and gene expression noise.

Study of voxel size effects

To assess the effects of voxel size on the results of our model, we performed a mesh refinement study. The results are shown in S4 Fig. We ran our model for three different crowdedness parameters. For each θ value, we ran our model for different mesh sizes to determine the extent of phenotype dependency on mesh resolution. As can be seen in S4 Fig, the kurtosis of the mRNA distributions converges as we refine the mesh. The kurtosis tends to near zero as the number of voxels tends to 1, as

expected since 1 voxel is a well-mixed simulation. This demonstrates the importance of the use of inhomogeneous stochastic simulation to capture the noise reduction by macromolecular crowding.

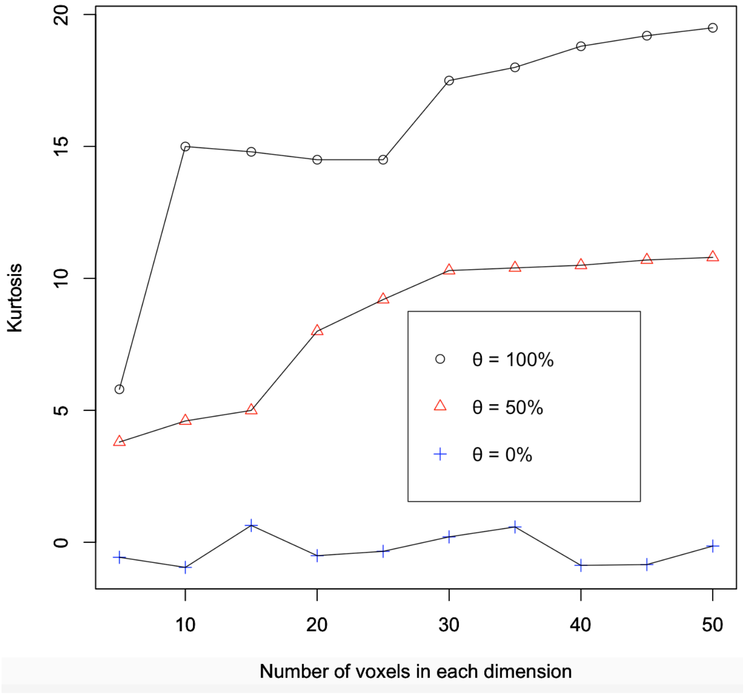

S4 Fig. Convergence study of modified NSM method. The kurtosis of the mRNA distributions converge as we refine the mesh; however, a coarser mesh is incapable of showing diffusion-limited gene expression noise reduction (δ_i = 0.1).

References

1. **Muramatsu N, Minton AP. Tracer diffusion of globular proteins in concentrated protein solutions. Proceedings of the National Academy of Sciences. 1988 May 1;85(9):2984-8.**
2. **Kaern M, Elston TC, Blake WJ, Collins JJ. Stochasticity in gene expression: from theories to phenotypes. Nature Reviews Genetics. 2005 Jun 1;6(6):451-64.**
